# Cyclic nucleotides and mitogen-activated protein kinases: regulation of simvastatin in platelet activation

**DOI:** 10.1186/1423-0127-17-45

**Published:** 2010-06-04

**Authors:** Ye-Ming Lee, Wei-Fan Chen, Duen-Suey Chou, Thanasekaran Jayakumar, Ssu-Yu Hou, Jie-Jen Lee, George Hsiao, Joen-Rong Sheu

**Affiliations:** 1 Department of Surgery, Hsinchu Mackay Memorial Hospital, Hsinchu; Mackay Medicine, Nursing and Management College, Taipei, Taiwan; 2 Department of Pharmacology, Taipei Medical University, Taipei, Taiwan; 3 Graduate Institute of Medical Sciences, Taipei Medical University, Taipei, Taiwan

## Abstract

**Background:**

3-Hydroxy-3-methyl-glutaryl coenzyme A (HMG-CoA) reductase inhibitors (statins) have been widely used to reduce cardiovascular risk. These statins (i.e., simvastatin) may exert other effects besides from their cholesterol-lowering actions, including inhibition of platelet activation. Platelet activation is relevant to a variety of coronary heart diseases. Although the inhibitory effect of simvastatin in platelet activation has been studied; the detailed signal transductions by which simvastatin inhibit platelet activation has not yet been completely resolved.

**Methods:**

The aim of this study was to systematically examine the detailed mechanisms of simvastatin in preventing platelet activation. Platelet aggregation, flow cytometric analysis, immunoblotting, and electron spin resonance studies were used to assess the antiplatelet activity of simvastatin.

**Results:**

Simvastatin (20-50 μM) exhibited more-potent activity of inhibiting platelet aggregation stimulated by collagen than other agonists (i.e., thrombin). Simvastatin inhibited collagen-stimulated platelet activation accompanied by [Ca^2+^]i mobilization, thromboxane A_2 _(TxA_2_) formation, and phospholipase C (PLC)γ2, protein kinase C (PKC), and mitogen-activated protein kinases (i.e., p38 MAPK, JNKs) phosphorylation in washed platelets. Simvastatin obviously increased both cyclic AMP and cyclic GMP levels. Simvastatin markedly increased NO release, vasodilator-stimulated phosphoprotein (VASP) phosphorylation, and endothelial nitric oxide synthase (eNOS) expression. SQ22536, an inhibitor of adenylate cyclase, markedly reversed the simvastatin-mediated inhibitory effects on platelet aggregation, PLCγ2 and p38 MAPK phosphorylation, and simvastatin-mediated stimulatory effects on VASP and eNOS phosphorylation.

**Conclusion:**

The most important findings of this study demonstrate for the first time that inhibitory effect of simvastatin in platelet activation may involve activation of the cyclic AMP-eNOS/NO-cyclic GMP pathway, resulting in inhibition of the PLCγ2-PKC-p38 MAPK-TxA_2 _cascade, and finally inhibition of platelet aggregation.

## Background

A high incidence of atherosclerosis and thrombotic complications has been associated with hypercholesterolemia. Blood cholesterol levels are of fundamental importance in the pathogenesis of coronary artery diseases (CAD). Elevations of low-density lipoprotein (LDL) levels are not only linked to an increased risk for atherosclerosis but may also exert prothrombotic effects via platelet activation [1]. The effectiveness of 3-hydroxy-3-methyl-glutaryl coenzyme A (HMG-CoA) reductase inhibitors (statins) in the prevention of CAD is ascribed not only to reduced cholesterol levels [2,3], but also to a number of additional effects, including the stabilization of atherosclerotic plaque, improved endothelial function, enhanced fibrinolysis, and antithrombotic effects [3-5]. Although many studies have demonstrated that statins have antiplatelet activity in hypercholesterolemic patients and animals [6], the inhibition of platelet-dependent thrombus formation in hypercholesterolemia may not correlate with the lipid-lowering effects, suggesting that these statins may exert another effect besides from their cholesterol-lowering actions.

Inhibition of the thromboxane B_2 _formation or changing cholesterol content on platelet membrane by statins has been reported [7,8]. Recently, Chou *et al*. [6] also suggested that enhanced nitric oxide (NO) and cyclic GMP formation of simvastatin (20-80 μM) may be involved in the inhibitory effects on platelet aggregation. The antiplatelet activity of simvastatin in platelets has been studied; however, the detailed signal transduction mechanism by which simvastatin inhibits platelet activation has not yet been completely resolved. We therefore systematically examined the cellular signal events associated with simvastatin-inhibited platelet activation in the present study.

## Methods

### Materials

Collagen (type I), luciferin-luciferase, phorbol-12, 13-dibutyrate (PDBu), 5,5-dimethyl-1 pyrroline N-oxide (DMPO), SQ22536, ODQ, arachidonic acid (AA), prostaglandin E_1 _(PGE_1_), nitroglycerin, and thrombin were purchased from Sigma Chem. (St Louis, MO); Fura 2-AM and fluorescein iso-thiocyanate (FITC) were from Molecular Probe (Eugene, OR); the thromboxane B_2 _enzyme immunoassay (EIA) kit was from Cayman (Ann Arbor, MI); the anti-vasodilator-stimulated phosphoprotein (VASP Ser^157^) monoclonal antibody (mAb) was from Calbiochem (San Diego, CA); the anti-phospho-p38 mitogen-activated protein kinase (MAPK) Ser^182 ^mAb was from Santa Cruz (Santa Cruz, CA); the anti-p38 MAPK and anti-phospho-c-Jun N-terminal kinase (JNK) (Thr^183^/Tyr^185^) mAbs, anti-phospholipase Cγ2 (PLCγ2), anti-phospho (Tyr^759^) PLCγ2 mAbs, and the anti-phospho-p44/p42 extracellular signal-regulated kinase (ERK) (Thr^202^/Tyr^204^) polyclonal antibody (pAb) were from Cell Signaling (Beverly, MA); the anti-α-tubulin mAb was from NeoMarkers (Fremont, CA); and the Hybond-P PVDF membrane, ECL Western blotting detection reagent and analysis system, horseradish peroxidase (HRP)-conjugated donkey anti-rabbit IgG, and sheep anti-mouse IgG were from Amersham (Buckinghamshire, UK). Cyclic AMP and cyclic GMP EIA kits were purchased from Cayman (Ann Arbor, MI). Simvastatin was dissolved in 0.5% dimethyl sulfoxide (DMSO) and stored at 4°C until used.

### Platelet aggregation

Human platelet suspensions were prepared as previously described [9]. This study was approved by the *Institutional Review Board of Taipei Medical University *and conformed to the principles outlined in the *Helsinki Declaration*, and all human volunteers provided informed consent. In brief, blood was collected from healthy human volunteers who had taken no medicine during the preceding 2 weeks, and was mixed with acid/citrate/glucose (9:1:1, v/v). After centrifugation, the supernatant (platelet-rich plasma; PRP) was supplemented with prostaglandin E_1 _(PGE_1_) (0.5 μM) and heparin (6.4 IU/ml). The washed platelets were finally suspended in Tyrode's solution containing bovine serum albumin (BSA) (3.5 mg/ml). The final concentration of Ca^2+ ^in Tyrode's solution was 1 mM.

A turbidimetric method was applied to measure platelet aggregation [9], using a Lumi-Aggregometer (Payton, Scarborough, Ontario, Canada). Platelet suspensions (0.4 ml) were preincubated with various concentrations of simvastatin or an isovolumetric solvent control (0.5% DMSO) for 3 min before the addition of agonists. The reaction was allowed to proceed for 6 min, and the extent of aggregation was expressed in light-transmission units. When measuring ATP release, 20 μl of a luciferin/luciferase mixture was added 1 min before the addition of agonists, and ATP release was compared to that of the control.

### Measurement of cyclic AMP and cyclic GMP formations

Platelet suspensions (3.6 × 10^8^/ml) were incubated with isovolumetric solvent control (0.5% DMSO), nitroglycerin (10 μM), PGE_1 _(10 μM), or simvastatin (30 and 50 μM) for 6 min. The incubation was stopped by the addition of EDTA (5 mM), and the solution was immediately boiled for 5 min. Fifty microliters of the supernatant was used to determine the cyclic AMP and cyclic GMP contents with EIA kits following acetylation of the samples as described by the manufacturer.

### Flow cytometric analysis

Triflavin, an α_IIb_β_3 _integrin antagonist, was prepared as previously described [10]. Fluorescence-conjugated triflavin was prepared as previously described [10]. Platelet suspensions (3.6 × 10^8^/ml) were preincubated with simvastatin (30 and 50 μM) or a solvent control for 3 min, followed by the addition of 2 μl of FITC-triflavin (2 μg/ml). The suspensions were then assayed for fluorescein-labeled platelets using a flow cytometer (Beckman Coulter, Miami, FL). Data were collected from 50,000 platelets per experimental group, and the platelets were identified on the basis of their characteristic forward and orthogonal light-scattering profile. All experiments were repeated at least four times to ensure reproducibility.

### Measurement of platelet [Ca^2+^]i by Fura 2-AM fluorescence

Citrated whole blood was centrifuged at 120 *g *for 10 min. The supernatant was incubated with Fura 2-AM (5 μM) for 1 h. Human platelets were then prepared as described above. Finally, the external Ca^2+ ^concentration of the platelet suspensions was adjusted to 1 mM. The [Ca^2+^]i rise was measured using a fluorescence spectrophotometer (CAF 110, Jasco, Tokyo, Japan) with excitation wavelengths of 340 and 380 nm, and an emission wavelength of 500 nm [9].

### Measurement of thromboxane B_2 _formation

Platelet suspensions (3.6 × 10^8^/ml) were preincubated with simvastatin (30 and 50 μM) or solvent control for 3 min before the addition of collagen (1 μg/ml). Six minutes after the addition of agonists, 2 mM EDTA and 50 μM indomethacin were added to the suspensions. The thromboxane B_2 _(TxB_2_) levels of the supernatants were measured using an EIA kit.

### Immunoblotting study

Washed platelets (1.2 × 10^9^/ml) were preincubated with simvastatin (30 and 50 μM) or a solvent control for 3 min, followed by the addition of agonists to trigger platelet activation. The reaction was stopped, and platelets were immediately re-suspended in 200 μl of lysis buffer. Samples containing 80 μg of protein were separated by SDS-PAGE (12%); the proteins were electrotransferred by semidry transfer (Bio-Rad, Hercules, CA). Blots were blocked with TBST (10 mM Tris-base, 100 mM NaCl, and 0.01% Tween 20) containing 5% BSA for 1 h and then probed with various primary antibodies. Membranes were incubated with HRP-linked anti-mouse IgG or anti-rabbit IgG (diluted 1: 3000 in TBST) for 1 h. Immunoreactive bands were detected by an enhanced chemiluminescence (ECL) system. The bar graph depicts the ratios of quantitative results obtained by scanning reactive bands and quantifying the optical density using videodensitometry (Bio-profil; Biolight Windows Application V2000.01; Vilber Lourmat, France).

### Estimation of nitrate formation

NO was assayed in platelet suspensions as previously described [10]. In brief, platelet suspensions (1.2 × 10^9^/ml) were preincubated with PGE_1 _(10 μM) or simvastatin (30 and 50 μM) for 3 min, followed by centrifugation. The amount of nitrate in the platelet suspensions (10 μl) was measured by adding a reducing agent to the purge vessel to convert nitrate to NO which was stripped from the suspensions by purging with helium gas. The NO was then drawn into a Sievers Nitric Oxide Analyzer (Sievers 280 NOA, Sievers, Boulder, CO). Nitrate concentrations were calculated by comparison with standard solutions of sodium nitrate.

### Measurement of hydroxyl radical by electron spin resonance (ESR) spectrometry

The ESR method used a Bruker EMX ESR spectrometer as described previously [11]. In brief, platelet suspensions (3.6 × 10^8^/ml) were preincubated with simvastatin (30 and 50 μM) or solvent control for 3 min before the addition of collagen (1 μg/ml). The reaction was allowed to proceed for 5 min, followed by the addition of DMPO (100 μM) for the ESR study. The rate of hydroxyl radical-scavenging activity is defined by the following equation: inhibition rate = 1-[signal height (simvastatin)/signal height (solvent control)] [11].

### Statistical analysis

The experimental results are expressed as the means ± S.E.M. and are accompanied by the number of observations. The experiments were assessed by the method of analysis of variance (ANOVA). If this analysis indicated significant differences among group means, then each group was compared using the Newman-Keuls method. *P *< 0.05 was considered statistically significant.

## Results

### Effects of simvastatin on platelet aggregation, α_IIb_β_3 _integrin conformational change, and [Ca^2+^]i mobilization in human platelets

**Figure 1:**
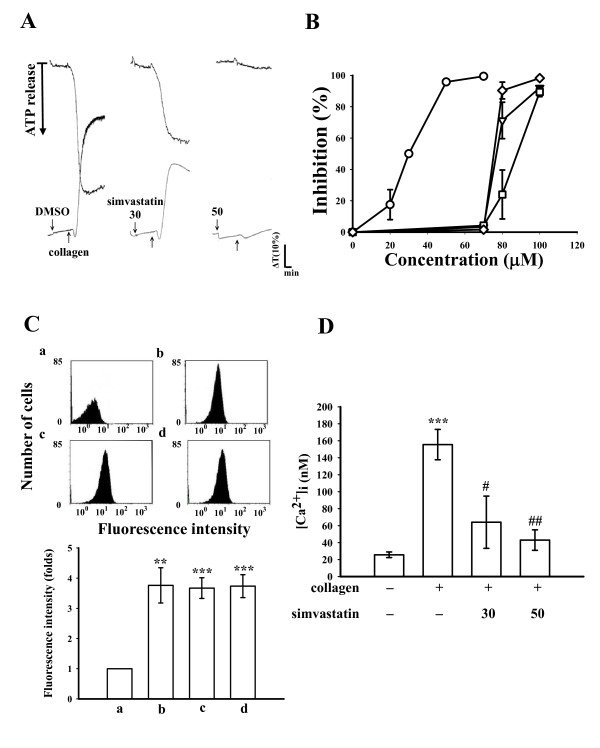
**Effects of simvastatin on the inhibition of (A and B) platelet aggregation, (C) FITC-triflavin binding to the α_IIb_β_3 _integrin and (D) [Ca^2+^]i mobilization in activated platelets**. Washed platelets (3.6 × 10^8^/ml) were preincubated with simvastatin (10-100 μM) or 0.5% DMSO, followed by the addition of collagen (1 μg/ml, open circle), U46619 (1 μM, upside down open triangle), thrombin (0.02 U/ml, open square) or arachidonic acid (60 μM, open diamond) to trigger platelet aggregation (A and B) and the ATP-release reaction (A, upper tracings) or (D) [Ca^2+^]i mobilization. (C) The solid line represents the fluorescence profiles of FITC-triflavin (2 μg/ml) (a) with or (b) without EDTA (5 mM); or pretreatment of simvastatin (c) (30 μM) and (d) (50 μM), followed by the addition of FITC-triflavin (2 μg/ml). Data are presented as the means ± S.E.M. (*n *= 4); ***P < 0.001, compared to the control group; ^#^*P *< 0.05 and ^##^*P *< 0.01, compared to the collagen group.

Simvastatin (20-70 μM) exhibited potent activity of inhibiting platelet aggregation and the ATP-release reaction stimulated by collagen (1 μg/ml, open circle). It also significantly inhibited platelet aggregation stimulated by thrombin (0.02 U/ml, open square), AA (60 μM, open diamond) or U46619 (1 μM, open triangle), a prostaglandin endoperoxide at higher concentrations (70-100 μM) (Fig. 1A and 1B). The IC_50 _value of simvastatin for platelet aggregation induced by collagen was approximately 30 μM. The solvent control (0.5% DMSO) did not significantly affect platelet aggregation stimulated by agonists in washed platelets (Fig. 1A). When platelets were preincubated with a higher concentration of simvastatin (200 μM) or 0.5% DMSO for 10 min, followed by two washes with Tyrode's solution, there were no significant differences between the aggregation curves of either platelet preparations stimulated by collagen (1 μg/ml), indicating that the effect of simvastatin on inhibition of platelet aggregation occurs in a reversible manner (data not shown). In subsequent experiments, we used collagen as an agonist to explore the inhibitory mechanisms of simvastatin in platelet activation.

Triflavin is an Arg-Gly-Asp-containing antiplatelet peptide purified from *Trimeresurus flavoviridis *snake venom [10]. Triflavin inhibits platelet aggregation through direct interference with fibrinogen binding to the α_IIb_β_3 _integrin [10]. There is now a multitude of evidence suggesting that the binding of fibrinogen to the α_IIb_β_3 _integrin is the final common pathway for agonist-induced platelet aggregation. Therefore, we further evaluated whether or not simvastatin directly binds to the platelet α_IIb_β_3 _integrin, leading to interruption of platelet aggregation induced by collagen. In this study, the relative intensity of the fluorescence of FITC-triflavin (2 μg/ml) bound directly to collagen (1 μg/ml)-activated platelets was relatively higher than that of negative control (in the presence of 5 mM EDTA) (a, 1.4 ± 0.2; b, 4.8 ± 0.4) (Fig. 1C). Simvastatin (30 and 50 μM) did not significantly affect FITC-triflavin binding to the α_IIb_β_3 _integrin in platelet suspensions (c, 4.8 ± 0.1; d, 4.9 ± 0.1) (Fig. 1C), indicating that the inhibitory effect of simvastatin on platelet aggregation does not involve binding to the platelet α_IIb_β_3 _integrin.

Free cytoplasmic Ca^+2 ^concentrations in human platelets were measured by the Fura 2-AM loading method. As shown in Figure 1D, collagen (1 μg/ml) evoked a marked increase in [Ca^2+^]i, and this increase was markedly inhibited in the presence of simvastatin (30 μM, 60.9 ± 17.0%; 50 μM, 72.1 ± 7.9%).

### Effects of simvastatin on TxA_2_, PLCγ2, and PKC activation

**Figure 2:**
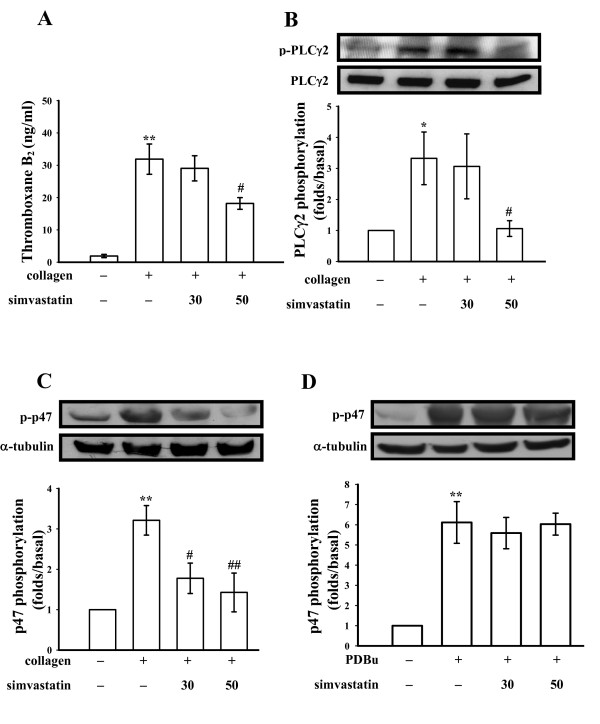
**Effects of simvastatin on (A) thromboxane B_2 _formation, (B) phospholipase Cγ2 and (C and D) PKC substrate (p47) phosphorylation in activated platelets**. Washed platelets were preincubated with simvastatin (30 and 50 μM) or 0.5% DMSO, followed by the addition of collagen (1 μg/ml) or PDBu (150 nM) to trigger platelet activation. Cells were collected, and subcellular extracts were analyzed for (A) thromboxane A_2 _formation, (B) phospholipase Cγ2 phosphorylation, and (C and D) phospho-PKC substrate (p-p47) as described in "Methods". Data are presented as the means ± S.E.M. (*n *= 4); *P < 0.05 and **P < 0.01, compared to the control group; ^#^*P *< 0.05 and ^##^*P *< 0.01, compared to the collagen group.

As shown in Figure 2A, resting platelets produced relatively little TxB_2 _compared to collagen-activated platelets. Simvastatin (30 and 50 μM) concentration-dependently inhibited TxB_2 _formation in platelets stimulated by collagen (1 μg/ml). PLC hydrolyzes phosphatidylinositol 4,5-bisphosphate (PIP_2_) to generate two secondary messengers: inositol 1,4,5-trisphosphate (IP_3_) and diacylglycerol (DAG) [12]. DAG activates PKC, inducing protein phosphorylation (p47) and ATP release. Phosphorylation is one of the key mechanisms regulating the activity of PLC. The immunoblotting analysis revealed that treatment with simvastatin markedly abolished the phosphorylation of PLCγ2 stimulated by collagen (Fig. 2B). Stimulation of platelets with a number of different agonists induced activation of PKC, which then phosphorylated p47 proteins. In this study, phosphorylation experiments were performed to examine the role of simvastatin in PKC activation in human platelets. When collagen (1 μg/ml) (Fig. 2C) or PDBu (150 nM) (Fig. 2D) was added to human platelets, a protein with an apparent of p47 was predominately phosphorylated compared to resting platelets. Simvastatin inhibited p47 phosphorylation stimulated by collagen but not by PDBu (Fig. 2C and 2D).

### Effect of simvastatin on collagen-induced MAPK phosphorylation

**Figure 3:**
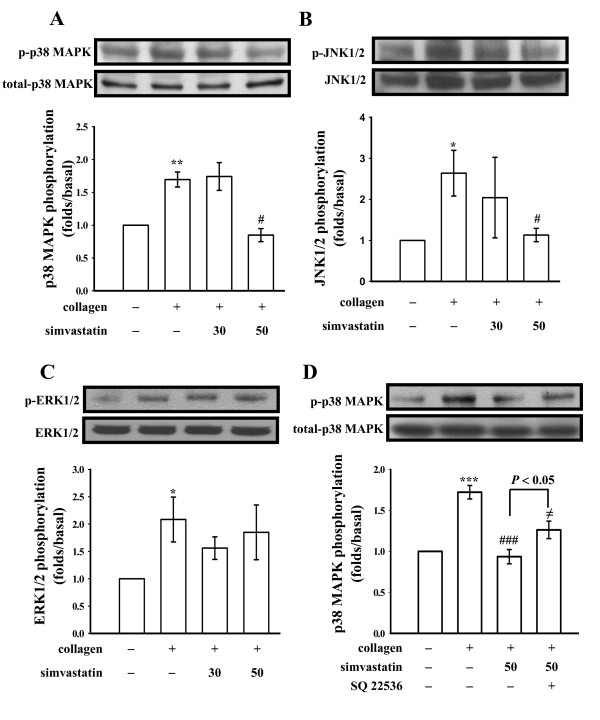
**Effects of simvastatin on (A and D) p38 MAPK, (B) JNKs, and (C) ERKs phosphorylation in activated platelets**. Washed platelets (1.2 × 10^9^/ml) were preincubated with simvastatin (30 and 50 μM) or 0.5% DMSO, followed by the addition of collagen (1 μg/ml) to trigger (A and D) p38 MAPK, (B) JNKs, and (C) ERKs phosphorylation. Data are presented as the means ± S.E.M. (*n *= 4); *P < 0.05 and **P < 0.01, compared to the control group; ^#^*P *< 0.05, compared to the collagen group.

To further investigate the inhibitory mechanisms of simvastatin in platelet activation stimulated by collagen, we further detected MAPK signaling molecules including p38 MAPK, JNKs, and ERKs. The immunoblotting analysis revealed that simvastatin (50 μM) inhibited p38 MAPK (Fig. 3A) and JNKs (Fig. 3B), but not ERKs (Fig. 3C) phosphorylation stimulated by collagen. In addition, in the presence of SQ22536 (100 μM), an inhibitor of adenylate cyclase, significantly reversed the simvastatin-mediated inhibition of p38 MAPK phosphorylation stimulated by collagen (Fig. 3D).

### Effects of simvastatin on cyclic nucleotides, nitrate formation and VASP phosphorylation

**Figure 4:**
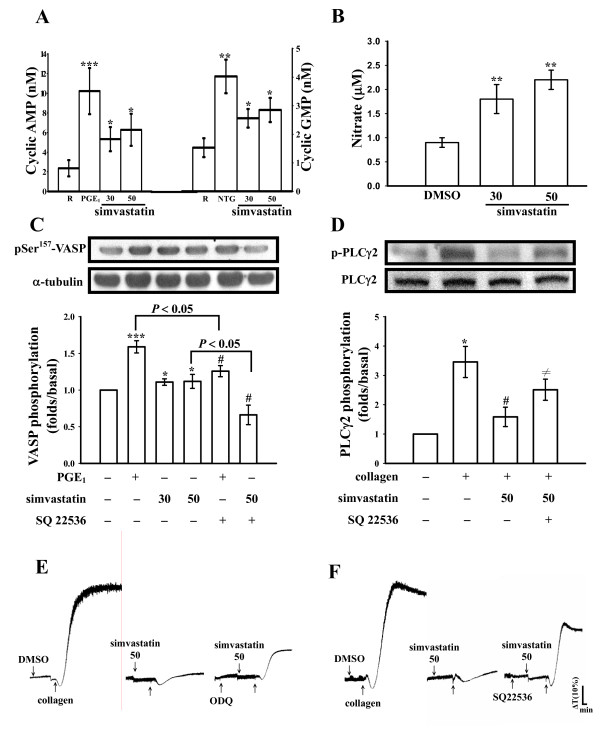
**Effects of simvastatin on (A) cyclic nucleotides (B) nitrate formations, (C) Ser^157^-vasodilator-stimulated phosphoprotein (VASP) and (D) phospholipase Cγ2 phosphorylation as well as (E and F) platelet aggregation in the presence of inhibitors of cyclic nucleotides in washed platelets**. Platelets were incubated with prostaglandin E_1 _(PGE_1_, 10 μM), nitroglycerin (NTG, 10 μM), simvastatin (30 and 50 μM), or 0.5% DMSO. Cells were collected, and subcellular extracts were analyzed for (A) cyclic nucleotides, (B) nitrate formations (C) Ser^157^-VASP, and (D) phospholipase Cγ2 phosphorylations as described in "Methods". For platelet aggregation study, washed platelets were preincubated with simvastatin (50 μM) in the absence or presence of (E) ODQ (20 μM) or (F) SQ22536 (100 μM), followed by the addition of collagen (1 μg/ml). Data are presented as the means ± S.E.M. (*n *= 3-4); *P < 0.05, **P < 0.01, and ***P < 0.001, compared to the control group; ^#^*P *< 0.05, compared to the without SQ22536 groups. ^≠^*P *< 0.05, compared to the collagen plus simvastatin group. The profiles (E and F) are representative examples of four similar experiments.

The level of cyclic AMP in unstimulated platelets was less, the addition of PGE_1 _(10 μM) markedly increased approximately 4.3-fold of cyclic AMP level compared with the resting group (Fig. 4A). Simvastatin (30 and 50 μM) significantly increased the cyclic AMP levels in human platelets (30 μM, 5.3 ± 1.2 nM; 50 μM, 6.3 ± 1.6 nM; *n *= 3) (Fig. 4A). We also performed a similar study measuring the cyclic GMP response. The level of cyclic GMP in unstimulated platelets was about 1.5 ± 0.3 nM, but when nitroglycerin (NTG, 10 μM) was added to the platelet suspensions, the cyclic GMP level markedly increased from the resting level to 4.0 ± 0.6 nM (*n *= 3) (Fig. 4A). The addition of simvastatin (30 and 50 μM) resulted in significant increases in platelet cyclic GMP levels (30 μM, 2.6 ± 0.3 nM; 50 μM, 2.9 ± 0.4 nM; *n *= 3) (Fig. 4A). NO was quantified using a sensitive and specific ozone redox-chemiluminescence detector. As shown in Figure 4B, simvastatin (30 and 50 μM) concentration-dependently increased nitrate production after incubation with washed platelets (Fig. 4B). It was demonstrated that cyclic nucleotides can induce VASP Ser^157 ^phosphorylation in human platelets [13]. In this study, PGE_1 _(10 μM) and simvastatin (30 and 50 μM) markedly induced VASP Ser^157 ^phosphorylation (Fig. 4C). SQ22536 (100 μM) significantly inhibited the phosphorylation stimulated by both PGE_1 _(10 μM) and simvastatin (50 μM) (Fig. 4C). Furthermore, SQ22536 (100 μM) obviously reversed the simvastatin (50 μM)-mediated inhibitory effect of PLCγ2 phosphorylation stimulated by collagen (Fig. 4D). On the other hand, pretreatment with SQ22536 (100 μM) or ODQ (20 μM), an inhibitor of guanylate cyclase, significantly reversed the simvastatin (50 μM)-mediated inhibition of platelet aggregation stimulated by collagen (Fig. 4E and 4F). These results indicate that simvastatin inhibits platelet aggregation, al least in part, via a cyclic nucleotides-dependent pathway.

### Effects of simvastatin on eNOS phosphorylation and hydroxyl radical formation

**Figure 5:**
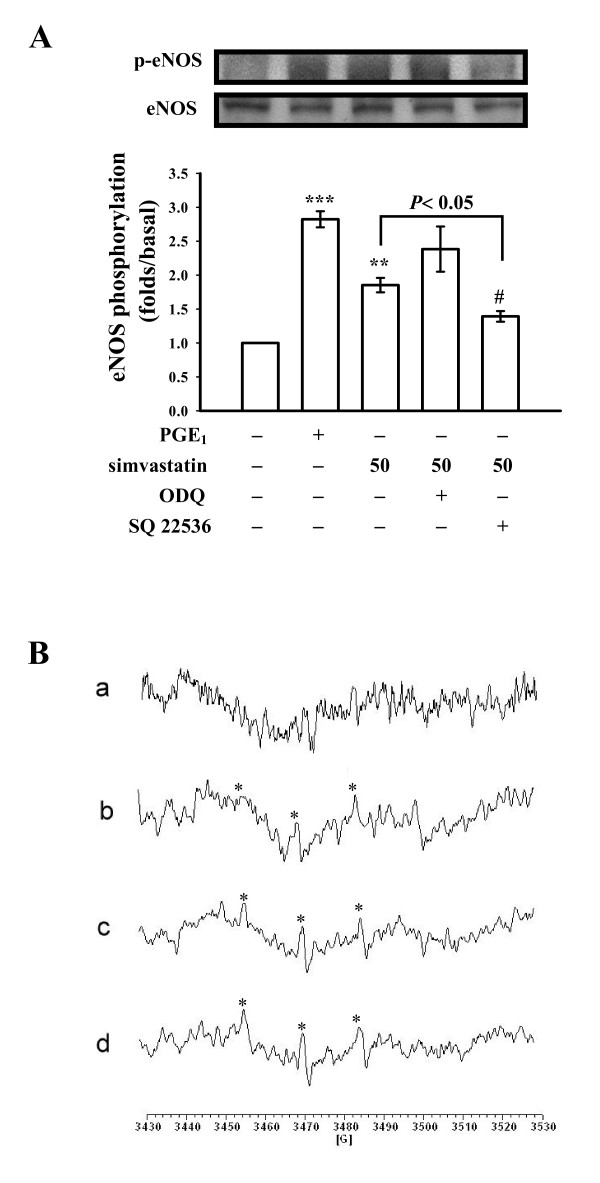
**Effects of simvastatin on (A) endothelial nitric oxide synthase (eNOS) phosphorylation and (B) hydroxyl radical (OH^•^) formation in activated platelets**. (A) Platelets were incubated with prostaglandin E_1 _(PGE_1_, 10 μM), simvastatin (30 and 50 μM), or 0.5% DMSO in the absence or presence of SQ22536 (100 μM) or ODQ (20 μM) as described in "Methods". Cells were collected, and subcellular extracts were analyzed for eNOS phosphorylation. Data are presented as the means ± S.E.M. (*n *= 4); **P < 0.01 and ***P < 0.001, compared to the control group; ^#^*P *< 0.05, compared to the PGE_1 _group. (B) For the electron spin resonance (ESR) study, platelets were preincubated with (a) Tyrode's solution (resting group), (b) a solvent control (0.5% DMSO), or simvastatin (30 and 50 μM), followed by the addition of collagen (1 μg/ml) to trigger platelet activation. Spectra are representative examples of four similar experiments. Asterisk (*) indicates the formation of hydroxyl radical.

Endothelial nitric oxide synthase (eNOS) phosphorylation was markedly activated by both PGE_1 _(10 μM) and simvastatin (50 μM) (Fig. 5A). The simvastatin-activated eNOS phosphorylation was significantly reversed in the presence of SQ22536 (100 μM) but not by ODQ (20 μM), indicating that cyclic AMP plays an up-regulator in simvastatin-mediated eNOS phosphorylation in human platelets (Fig. 5A). On the other hand, a typical ESR signal of hydroxyl radical (OH^•^) formation was induced in collagen (1 μg/ml)-activated platelets compared to resting platelets (Fig. 5B, a and 5b); pretreatment with simvastatin (30 and 50 μM) did not significantly reduce hydroxyl radical formation stimulated by collagen (Fig. 5B, c and 5d). The antioxidant, catalase (1000 U/ml), markedly suppressed hydroxyl radical formation by about 78% (data not shown).

## Discussion

This study demonstrates for the first time that simvastatin inhibits platelet activation via a novel pathway: activation of cyclic AMP-eNOS/NO-cyclic GMP and inhibition of MAPK phosphorylation (i.e., p38 MAPK and JNKs) in washed platelets. Simvastatin exhibited more-potent activity at inhibiting collagen-induced platelet aggregation than other agonists. For the clinical therapy, the approved starting dose of simvastatin for most patients is 20 mg, and the maximal dose is 80 mg. In this study, the concentrations of simvastatin were employed at 30 and 50 μM, and the concentration of collagen was used at 1 μg/ml to trigger platelet aggregation. In general, concentrations of collagen were employed for platelet aggregation of from 0.1 to 5 μg/ml. In an attempt to elucidate the detailed mechanisms of pharmacological interest, we used a higher concentration (1 μg/ml) of collagen to induce a more-pronounced signal transduction in platelets (i.e., MAPKs and PKC etc.). Therefore, the pharmacological concentrations (30-50 μM) of simvastatin employed to inhibit platelet aggregation *in vitro *are reasonable higher than that of blood concentrations obtained during a simvastatin regimen *in vivo*. However, the concentration employed is closely to that of other *in vitro *studies (20-80 μM) [6,14,15].

Stimulation of platelets by agonists (i.e., collagen) causes marked alterations in phospholipid metabolism. The activation of PLC results in the degradation of phosphoinositides, notably, phosphatidylinositol 4,5-bisphosphate (PI4,5-P_2_), resulting in the production of the second messengers, inositol 1,4,5-trisphosphate (IP_3_) and DAG [16]. DAG activates PKC, inducing protein phosphorylation (p47). PKC activation represents a strategy adopted by cells to allow selected responses to specific activating signals in distinct cellular compartments [17]. Phosphoinositide-specific PLC is a key enzyme in signal transduction [18]. There are six major families of PLC enzymes which consist of at least 13 PLC isoforms [18]. PLCγ2 is involved in antigen-dependent signaling in B cells and collagen-dependent signaling in platelets [19]. In this study, both PLCγ2 phosphorylation and PKC activation stimulated by collagen were inhibited by simvastatin, suggesting that simvastatin-mediated antiplatelet activity is involved in inhibition of the PLCγ2-PKC signal pathway. Simvastatin had no direct effect on PKC activation, as it did not inhibit PDBu-induced PKC activation (Fig. 2D) or platelet aggregation (data not shown). In addition, collagen-induced TxB_2 _formation, a stable metabolite of TxA_2_, was markedly inhibited by simvastatin. TxA_2 _is important for collagen-induced platelet aggregation. This may explain the more-potent activity of simvastatin in inhibiting collagen-induced platelet aggregation than other agonists (thrombin and U46619).

MAPKs consist of three major subgroups. Growth factors preferentially activate ERKs (p44 ERK1 and p42 ERK2), which are involved in proliferation, adhesion, and cell progression [20], whereas p38 MAPK and JNKs (p46 JNK1 and p54 JNK2) are more responsive to stress, and appear to be involved in apoptosis [20]. ERKs, JNKs, and p38 MAPK have been identified in platelets [20]. The roles of JNKs and ERKs in physiopathology are unclear, but they have been suggested to be suppressors of α_IIb_β_3 _integrin activation or negative regulators of platelet activation [21]. On the other hand, p38 MAPK provides a crucial signal as a downstream effector of PKC which is necessary for aggregation caused by collagen [22]. Among the numerous downstream targets of p38 MAPK, the most physiologically relevant one in platelets is cytosolic phospholipase A_2 _(cPLA_2_). p38 MAPK is essential for the stimulation of cPLA_2_, which catalyzes AA release to produce TxA_2 _[23]; thus, p38 MAPK appears to provide a TxA_2_-dependent platelet aggregation pathway. Simvastatin significantly inhibits TxA_2 _formation, at least in part, via inhibition of p38 MAPK phosphorylation.

Activation of human platelets is inhibited by two intracellular pathways regulated by either cyclic AMP or cyclic GMP. The importance of cyclic AMP and cyclic GMP in modulating platelet reactivity is well established [24]. In addition to inhibiting most platelet responses, elevated levels of cyclic AMP or/and cyclic GMP decrease intracellular Ca^2+ ^concentrations by the uptake of Ca^2+ ^into the dense tubular system (DTS) which negatively affects the action of PLC and/or PKC [24]. Therefore, cyclic AMP and cyclic GMP act synergistically to inhibit platelet aggregation. In this study, simvastatin obviously increased the levels of both cyclic AMP and cyclic GMP in human platelets. Platelets produce NO in smaller amounts than do endothelial cells [25]. Most cellular actions of NO occur via stimulation of intracellular guanylate cyclase, leading to increases in cyclic GMP. Both the inducible NOS (iNOS) and eNOS isoforms have been described in platelets, but eNOS is predominant [25]. Simvastatin (80 μM) has been reported to induce NO release and stimulate eNOS activity in rabbit platelets [6]. In this study, SQ22536 markedly reversed simvastatin-mediated inhibition of platelet aggregation, PLCγ2, and p38 MAPK phosphorylation stimulated by collagen, and it also reversed the simvastatin-mediated activation of both eNOS and VASP phosphorylations. VASP is phosphorylated by cyclic nucleotide-dependent protein kinase in platelets, which plays important role in modulating actin filament dynamics and integrin activation [13]. In this study, simvastatin was found to stimulate eNOS phosphorylation, and this effect was reversed by SQ22536 but not by ODQ. This result is in accord with that of increased cyclic AMP stimulating eNOS activity and NO biosynthesis [26].

Reactive oxygen species (i.e., hydrogen peroxide and hydroxyl radicals) derived from platelet activation might amplify platelet reactivity during thrombus formation. Free radical species act as secondary messengers that increase cytosolic Ca^2+ ^during the initial phase of platelet activation processes, and PKC is involved in receptor-mediated free radical production in platelets [11]. The antiplatelet effect of simvastatin did not mediate by the free radical-scavenging activity in ESR experiment.

In conclusion, the most important findings of this study demonstrate for the first time that the antiplatelet activity of simvastatin may involve an increase of the cyclic AMP-eNOS/NO-cyclic GMP pathway, followed by inhibition of the PLCγ2-PKC-p38 MAPK-TxA_2 _cascade, thereby leading to inhibition of platelet aggregation. Hypercholesterolemic patients usually associate with a high incidence of atherosclerosis and thrombotic complications. This study provides a new insight of antiplatelet mechanisms of simvastatin to explain its clinical protective effect in CAD.

## Competing interests

The authors declare that they have no competing interests.

## Authors' contributions

YML and WFC carried out the platelet aggregation study and drafted the manuscript. DSC carried out the ESR study. TJ, SYH, and JJL carried out the immunoblotting study. GH performed the statistical analysis. JRS conceived of the study, and participated in the design and coordination, and collectively prepared the manuscript. All authors read and approved the final manuscript.
